# Itaconic acid mediates crosstalk between macrophage metabolism and peritoneal tumors

**DOI:** 10.1172/JCI99169

**Published:** 2018-07-30

**Authors:** Jonathan M. Weiss, Luke C. Davies, Megan Karwan, Lilia Ileva, Michelle K. Ozaki, Robert Y.S. Cheng, Lisa A. Ridnour, Christina M. Annunziata, David A. Wink, Daniel W. McVicar

**Affiliations:** 1 Cancer and Inflammation Program, Center for Cancer Research, National Cancer Institute (NCI) at Frederick, Frederick, Maryland, USA.; 2 Cardiff University, Division of Infection and Immunity, Cardiff, United Kingdom.; 3 Frederick National Laboratory for Cancer Research, Leidos Biomedical Research Inc., Frederick, Maryland, USA.; 4 Women’s Malignancies Branch, Center for Cancer Research (CCR), NCI, Bethesda, Maryland, USA.

**Keywords:** Metabolism, Oncology, Intermediary metabolism, Monocytes, Mouse models

## Abstract

Control of cellular metabolism is critical for efficient cell function, although little is known about the interplay between cell subset–specific metabolites in situ, especially in the tumor setting. Here, we determined how a macrophage-specific (Mϕ-specific) metabolite, itaconic acid, can regulate tumor progression in the peritoneum. We show that peritoneal tumors (B16 melanoma or ID8 ovarian carcinoma) elicited a fatty acid oxidation–mediated increase in oxidative phosphorylation (OXPHOS) and glycolysis in peritoneal tissue–resident macrophages (pResMϕ). Unbiased metabolomics identified itaconic acid, the product of immune-responsive gene 1–mediated (*Irg1*-mediated) catabolism of mitochondrial *cis*-aconitate, among the most highly upregulated metabolites in pResMϕ of tumor-bearing mice. Administration of lentivirally encoded *Irg1* shRNA significantly reduced peritoneal tumors. This resulted in reductions in OXPHOS and OXPHOS-driven production of ROS in pResMϕ and ROS-mediated MAPK activation in tumor cells. Our findings demonstrate that tumors profoundly alter pResMϕ metabolism, leading to the production of itaconic acid, which potentiates tumor growth. Monocytes isolated from ovarian carcinoma patients’ ascites fluid expressed significantly elevated levels of IRG1. Therefore, IRG1 in pResMϕ represents a potential therapeutic target for peritoneal tumors.

## Introduction

The peritoneal cavity is a frequent site of tumor development and metastasis. Ovarian epithelial cancer often spreads to the peritoneal cavity and is treated similarly to primary peritoneal cancer (1). The peritoneal cavity is also home to a heterogeneous population of peritoneal tissue–resident macrophages (pResMϕ) that arise from hematopoietic progenitors and play important roles in homeostasis and immune surveillance (2, 3). There is clearly the potential for extensive crosstalk between tumor cells and pResMϕ within the peritoneal cavity. We hypothesized that peritoneal tumors might profoundly influence the metabolism, and therefore the function, of pResMϕ and specifically sought to characterize those metabolic alterations that in turn regulate tumor progression.

Although Mϕ can mediate important antitumor responses, there is considerable evidence for their role in promoting the initiation, growth, and metastatic spread of many tumors. The ability of Mϕ to produce immunosuppressive cytokines and angiogenic factors as well as promote tumor growth has been well described (4). In the peritoneum, Mϕ depletion can reduce tumor progression, metastasis (5), and ascites fluid formation (6). Mϕ-targeted therapies are thus promising strategies for the treatment of ovarian carcinoma and peritoneal tumors.

Although tumor metabolism has been well studied, it is important to define the effects of host-tumor interactions on immune cell metabolism when metabolism-based therapies are used. Tumors have long been known to have high glycolytic rates, as they consume large amounts of glucose and release metabolic products of glycolysis. Glycolytic tumors have been proposed to metabolically restrict T cells, in part by outcompeting them for nutrients such as glucose (7). Moreover, tumor-derived metabolites, such as lactic acid, have been implicated in polarizing Mϕ toward a tumor-promoting phenotype, characterized by high levels of VEGF and arginase (8). Mϕ polarization is closely linked to metabolic programming, with alterations in key signaling pathways underlying distinct utilization modes of glucose metabolism (9–14). Classically activated Mϕ exhibit increases in glycolysis mediated through the AKT/mTOR/HIF1α pathway to regulate the inflammatory phenotype. In contrast, alternatively activated Mϕ have a vastly different metabolic and inflammatory profile promoted by increased oxidative phosphorylation (OXPHOS). Given that cellular metabolism is critical for the regulation of the Mϕ phenotype, tumor-mediated alterations in the expression of Mϕ metabolites clearly have the potential to underlie important differences in the effector function of pResMϕ.

Itaconate is among the most selectively and highly upregulated metabolites in classically activated Mϕ (13, 15, 16). Itaconate mediates antimicrobial functions in Mϕ by inhibiting isocitrate lyase, an enzyme of the glyoxylate shunt used as a bacterial survival mechanism (17). Recently, mitochondria-associated itaconate has emerged as an important metabolic regulator of Mϕ function by regulating the glycolytic pathway and promoting succinate accumulation during mitochondrial respiration (18, 19). Mammalian production of itaconate, or itaconic acid, is catalyzed by immune-responsive gene 1 (*Irg1*), which is transcriptionally regulated by IFN-γ, TNF-α, and LPS stimulation (20, 21). IRG1 catabolizes fatty acid–derived metabolites, which are necessary energy substrates for OXPHOS in Mϕ and OXPHOS-associated ROS production by mitochondria (14, 22–24). The IRG1 product itaconate thus links metabolism with proinflammatory processes and ROS production in Mϕ through alterations in fatty acid oxidation and OXPHOS.

ROS encompass a wide range of molecules such as hydrogen peroxide, superoxide, hydroxyl radicals, and peroxynitrite. In addition to serving as a critical component of Mϕ bactericidal activity, ROS production can have profound effects on inflammatory responses and tumor progression. ROS activate numerous transcription factors (e.g., NF-κB, HIF1α, STAT3, and AP-1), resulting in a complex association between ROS expression and tumor progression (25–27). Although ROS are a critical component of many antitumor responses, ROS expression has also been associated with the promotion of tumor cell growth and survival, in part through enhanced expression and activation of NF-κB and MAPK (26, 28, 29).

In this study, we demonstrate that itaconate is among the most highly upregulated metabolites in pResMϕ from tumor-bearing mice and that an increase in its levels plays an important role in tumor progression. Targeted blockade of tumor-induced itaconate in pResMϕ using a lentivirus-based shRNA approach demonstrates that itaconate promotes tumor growth via OXPHOS-driven ROS expression in resident Mϕ and concomitant ROS-mediated MAPK activation in tumor cells. Our data suggest that metabolic targeting of IRG1 and itaconate in Mϕ may be effective for the control of ROS levels and ROS-driven tumor progression. An advantage of our approach is the feasibility of specifically targeting resident Mϕ–associated itaconate and ROS for efficient control of peritoneal tumors.

## Results

### Resident Mϕ mediate protumor effects in the peritoneum.

**Figure 1:**
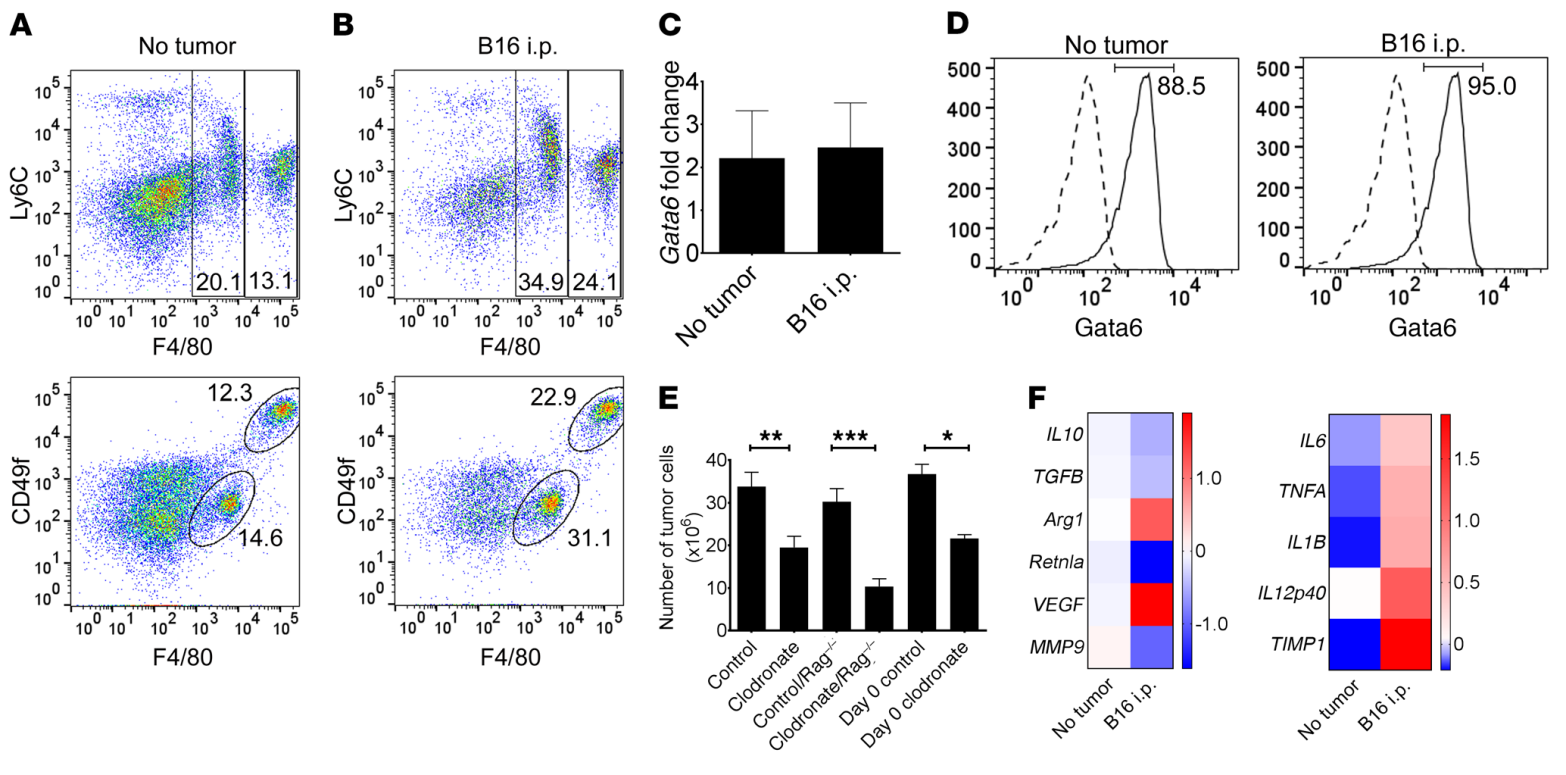
Characterization of peritoneal leukocytes from tumor-bearing hosts. Flow cytometric analysis was performed on the peritoneal lavage cells from (**A**) naive and (**B**) B16 tumor–bearing mice. Among F4/80-sorted leukocytes, Gata6 expression was evaluated by (**C**) qPCR and (**D**) intracellular flow cytometry. Flow cytometric plots are representative of 6 samples (2 experiments each consisting of triplicate samples). The dotted lines represent isotype control staining. (**E**) B16 tumor burden was quantified in WT or *Rag^–/–^* mice that received control or clodronate liposomes on day 2 (after tumor) or day 0 (before tumor) (*n* ≥5). *P < 0.05, **P < 0.01, and ***P < 0.001, by ANOVA with Tukey’s multiple comparisons test. (**F**) F4/80-sorted Mϕ were evaluated by qPCR for M2 (left) and M1 (right) prototypic gene expression. Triplicate samples were evaluated, with one of the no-tumor samples set to 1.0 as a reference point (heatmaps depict log_2_-transformed, relative-based results of gene expression; all genes shown were significantly altered in the B16 group to at least *P* < 0.05 as compared with the no-tumor group; unpaired Student’s *t* test). Data represent the mean ± SEM.

Hypothesizing that progressing tumors extensively interact with pResMϕ, we first compared macrophages (Mϕ) from naive and tumor-bearing mice. F4/80^+^ Mϕ from peritoneal lavages could be separated into 2 populations on the basis of their relative levels of F4/80 and CD49f expression (Figure 1A). The 2 Mϕ populations are consistent with larger (LPM) and smaller (SPM) pResMϕ populations that have been described previously (2, 30). The relative proportion of LPMs and SPMs was similar between control mice and mice bearing peritoneal tumors, although the overall frequency of F4/80^+^ Mϕ was increased in tumor-bearing mice (Figure 1B and Supplemental Figures 1, A–D; supplemental material available online with this article; https://doi.org/10.1172/JCI99169DS1), with the total number of leukocytes remaining similar between control and tumor-bearing mice. To further characterize pResMϕ, we evaluated their expression of Gata-binding factor 6 (Gata6), a transcription factor shown to be critical for their phenotype (31, 32). *Gata6* mRNA (Figure 1C) and protein expression levels were similar between pResMϕ from control and tumor-bearing mice (Figure 1D and Supplemental Figure 1E).

Numerous studies have ascribed a protumor phenotype for tumor-associated Mϕ (4). Consistently, we found that specific depletion of peritoneal Mϕ (>90% depletion) 2 days after tumor inoculation resulted in a significantly reduced tumor burden (Figure 1E). We obtained similar results using *Rag1^–/–^* mice, indicating that the protumor effects were independent of T and B lymphocytes. Moreover, the reductions in tumor burden were indistinguishable when the clodronate-depleting agent was administered prior to tumor inoculation (day 0), suggesting that the pResMϕ populations present in naive mice already have tumor-promoting potential. The protumor phenotype of resident Mϕ could not be attributed to a shift between pro- and antiinflammatory phenotypes. Although arginase 1 (*Arg1*) and *Vegf* increased, the expression of most prototypically protumor genes we evaluated was decreased in cells from tumor-bearing mice (Figure 1F, left), with a concomitant increase in all proinflammatory genes evaluated (Figure 1F, right). These data indicate that pResMϕ exhibit a tumor-promoting phenotype that cannot be ascribed to a generalized skewing in the expression of prototypic immunosuppressive and tumor-promoting molecules. Indeed, it has been suggested that classifying Mϕ as polarized and ascribing Mϕ function on the basis of pro- and antiinflammatory gene expression is an oversimplification and that consideration of gene function is more meaningful (33).

### The metabolism of resident Mϕ from tumor-bearing mice is increased as a result of fatty acid oxidation.

**Figure 2:**
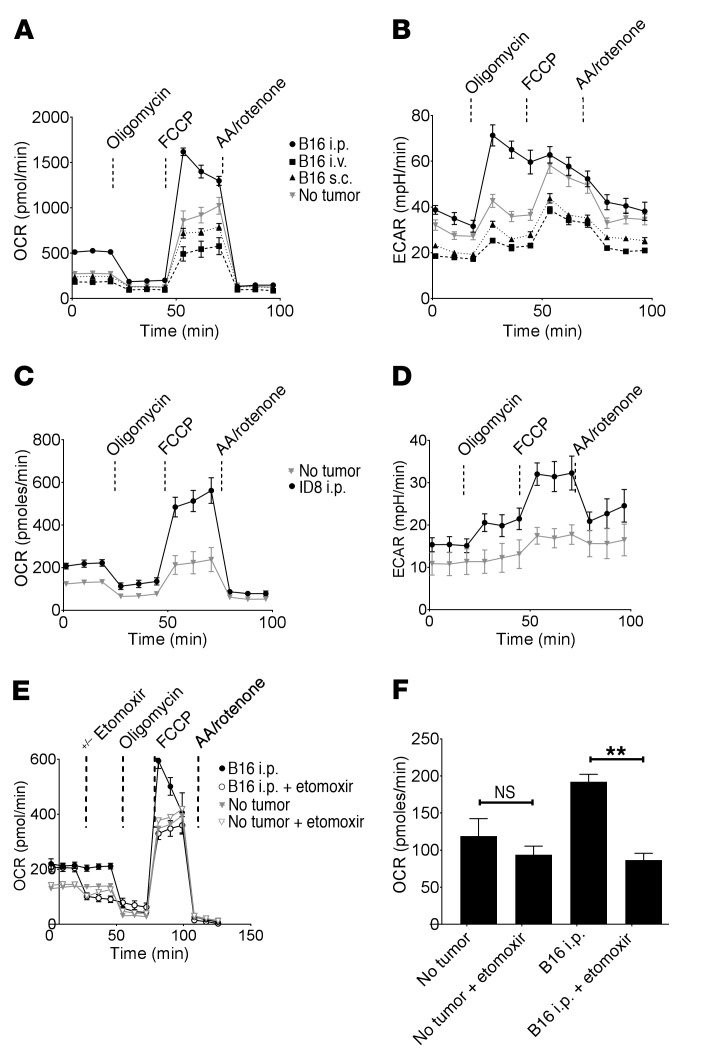
Tumors increase oxidative and glycolytic metabolism in pResMϕ. Extracellular flux analysis of F4/80-sorted pResMϕ from non–tumor-bearing control mice or mice inoculated with B16 via the indicated routes were analyzed. The (**A**) OCR and (**B**) ECAR were graphed over time as indicators of OXPHOS and glycolysis, respectively. Drugs were injected into the ports at the indicated time points. F4/80-sorted pResMϕ from no–tumor-bearing control mice or mice bearing ID8 ovarian carcinoma (day 47) were similarly evaluated for (**C**) cellular OCR and (**D**) ECAR. (**E**) OXPHOS of peritoneal Mϕ from control and B16 tumor–bearing mice were evaluated following injection of etomoxir into the first port at the indicated time point. (**F**) Basal OCRs for the treatment groups were graphed. **P < 0.01, by ANOVA with Tukey’s multiple comparisons test. All plots are representative of 3 experiments. Data represent the mean ± SEM. AA, Antimycin A.

We hypothesized that metabolic alterations in pResMϕ may underlie their protumor phenotype. We therefore evaluated the extracellular flux of pResMϕ isolated from control and tumor-bearing mice. pResMϕ from mice bearing peritoneal B16 tumors (i.p. inoculation) had dramatically elevated basal and maximal oxygen consumption rates (OCRs) (Figure 2A) and extracellular acidification rates (ECARs) (Figure 2B) as compared with those of control mice or mice inoculated with B16 tumors via distant routes (i.v. or s.c.). This suggests that increased pResMϕ metabolism is locally modulated by peritoneal tumors. We observed similar results using the ID8 ovarian carcinoma cell line (Figure 2, C and D). Cells can use diverse mechanisms to increase OXPHOS, however, it has been reported that antiinflammatory Mϕ use fatty acids to support OXPHOS (34). We therefore hypothesized that pResMϕ might use fatty acids to support their protumor phenotype and confirmed that fatty acid oxidation was an important component of tumor-mediated increases in OXPHOS, as treatment with etomoxir reduced basal OCRs to levels observed in non–tumor-bearing mice (Figure 2, E and F).

### Itaconic acid is the most highly upregulated metabolite in pResMϕ from tumor-bearing mice.

**Figure 3:**
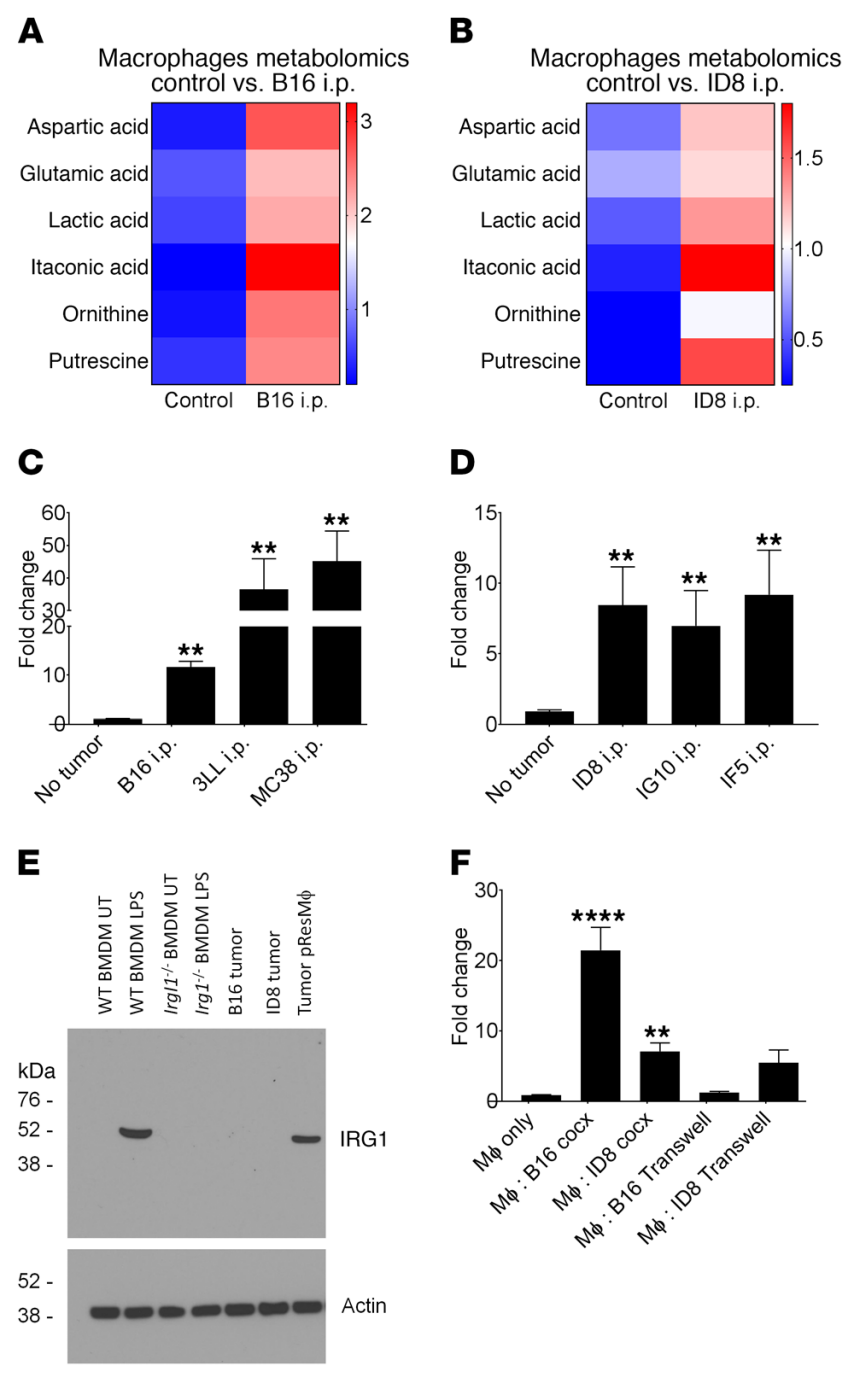
Irg1 and itaconic acid are upregulated in pResMϕ by peritoneal tumors. Unbiased metabolomic analysis was performed on F4/80-sorted pResMϕ from control and either (**A**) B16 melanoma– or (**B**) ID8 ovarian carcinoma–bearing mice. The mean values from at least 5 replicate samples were log_10_ transformed and plotted (*P* < 0.05 for all metabolites by unpaired Student’s *t* test). *Irg1* gene expression was evaluated by qPCR in F4/80-sorted pResMϕ from control mice and mice bearing either (**C**) day-9 B16 melanoma, 3LL, or MC38 tumors or (**D**) day-30 ID8, IG10, or IF5 ovarian carcinomas. Triplicate samples were evaluated, with one of the no-tumor control samples serving as the 1.0 relative reference point. **P < 0.01, by unpaired Student’s *t* test. (**E**) IRG1 protein levels in B16 and ID8 tumor lysates and pResMϕ purified from tumors were determined by Western blotting. Unstimulated or LPS-stimulated bone marrow–derived Mϕ from WT and *Irg1^–/–^* mice were used as controls. (**F**) pResMϕ were cocultured in vitro with the indicated tumor cells for 48 hours. *Irg1* expression was evaluated by qPCR (*n* = 3). cocx, co-culture. **P < 0.01 and ****P < 0.0001, by ANOVA with Tukey’s multiple comparisons test. Data represent the mean ± SEM.

Since Mϕ metabolism was significantly increased by tumors, we next investigated whether altered metabolite levels in pResMϕ are associated with tumor promotion. Using untargeted profiling of primary metabolites in purified pResMϕ, we identified several metabolites that were significantly upregulated in B16 and ID8 tumor-bearing mice as compared with naive mice (Figure 3, A and B, respectively). Among the most highly upregulated metabolites were lactic acid, previously implicated in Mϕ polarization (8), ornithine and polyamines such as putrescine, implicated in arginine metabolism and tumor promotion (35), and, somewhat surprisingly, itaconic acid, which was the most highly upregulated metabolite (>15-fold). Itaconate accumulation is catalyzed by the enzyme encoded by *Irg1* (20); accordingly, we found that *Irg1* mRNA expression was significantly upregulated in pResMϕ from mice bearing B16, 3LL, or MC38 tumors (Figure 3C) as well as in mice bearing ID8, IG10, or IF5 ovarian carcinomas (Figure 3D). Ingenuity Pathway Analysis (IPA) (QIAGEN; https://www.qiagenbioinformatics.com/products/ingenuitypathway-analysis) of our metabolomics data confirmed not only that itaconate was the most highly upregulated metabolite but that several saturated fatty acids were also significantly downregulated (Table 1), consistent with the association between itaconic acid production and fatty acid degradation (23). Western blotting confirmed IRG1 protein expression in pResMϕ from tumor-bearing mice, but IRG1 was not detected in B16 or ID8 tumor lysates, further confirming that the IRG1 expression in our system was due to the pResMϕ (Figure 3E). In vitro, we observed that *Irg1* expression was also upregulated in pResMϕ cocultured with B16 or ID8 tumor cells (Figure 3F). Interestingly, the ability of B16 tumor cells to induce *Irg1* expression in pResMϕ was lost when the tumor cells were separated from the Mϕ by a Transwell insert, suggesting that cell-cell contact is important for B16-mediated *Irg1* induction. In contrast, ID8 cells were still capable of inducing *Irg1* expression in Transwell conditions, indicating a distinct mechanism of *Irg1* induction for these tumor cells that involved soluble factor(s) (Figure 3F). These data suggest that tumors elicit an accumulation of itaconic acid as a result of *Irg1* expression in pResMϕ.

### Irg1 silencing in pResMϕ reduces peritoneal tumor burden.

**Figure 4:**
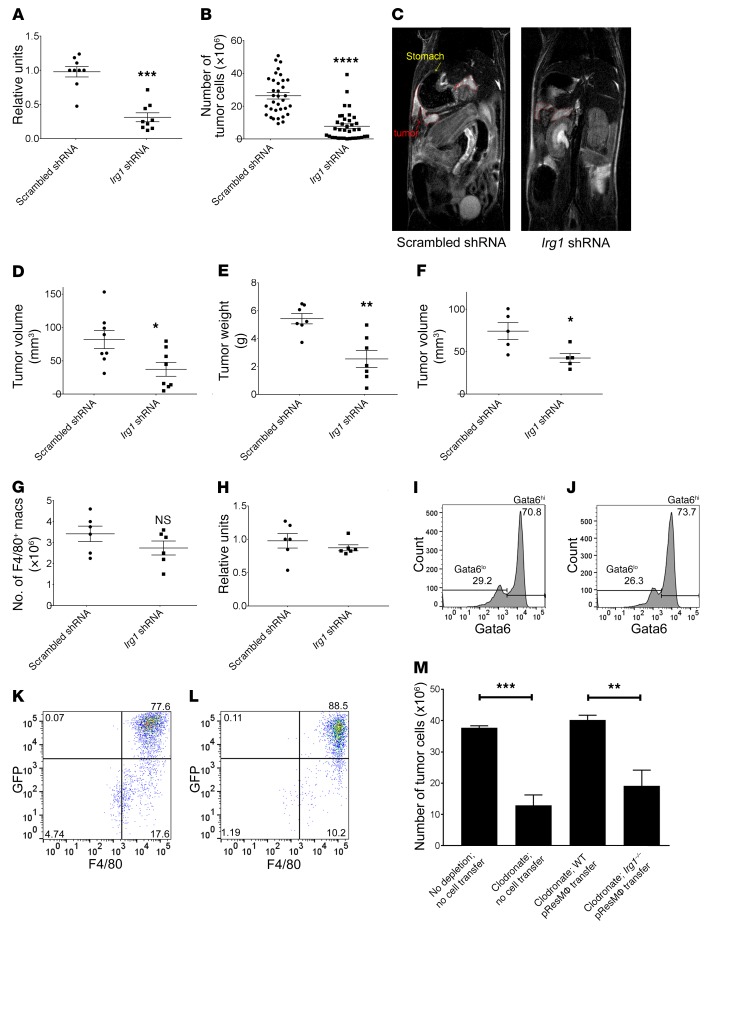
*Irg1* silencing in pResMϕ reduces peritoneal tumor burden. B16 tumor–bearing mice were treated with lentiviral shRNA (scrambled control or *Irg1* silencing). (**A**) Gene expression of *Irg1* was evaluated by qPCR (*n* = 9). (**B**) B16 tumor burden was quantitated and (**C**) evaluated by MRI of live tumor-bearing mice (MRI images are representative of 8 mice). (**D**) Tumor volumes were calculated by computing the tumor area on each plane and multiplying by the thickness of each slice (*n* = 8). The tumor weight (**E**) and volume (**F**) of ID8 ovarian carcinoma–bearing mice were measured (*n* ≥5). (**G**) Total numbers of F4/80^+^ pResMϕ were comparable among 6 scrambled shRNA and *Irg1* shRNA recipient mice. *Gata6* gene expression by qPCR (**H**) and protein levels were indistinguishable among scrambled (**I**) and *Irg1* (**J**) shRNA recipient mice. A similar uptake of lentiviral shRNA was confirmed by EGFP visualization in F4/80^+^ pResMϕ isolated from mice receiving scrambled (**K**) or *Irg1* (**L**) shRNA constructs. All FACS plots are representative of at least 6 mice per group. (**M**) Clodronate-depleted CD45.1 congenic mice (5 mice/group) were inoculated i.p. with peritoneal lavage cells from WT or *Irg1^–/–^* mice 1 day prior to tumor inoculation, and the B16 tumor burden was quantitated. Data represent the mean ± SEM. *P < 0.05, **P < 0.01, ***P < 0.001, and ****P < 0.0001, by Mann-Whitney *U* test.

To more directly delineate a possible role for itaconic acid in tumor progression, we used a lentiviral shRNA approach that specifically targets *Irg1* expression in pResMϕ (3, 32). Injection of *Irg1* shRNA i.p. resulted in a significant reduction of *Irg1* expression in F4/80-sorted pResMϕ (Figure 4A). Remarkably, *Irg1* shRNA treatment significantly reduced B16 tumor burden in the peritoneum, as determined by cell counting (Figure 4B) and MRI imaging of live tumor-bearing mice (Figure 4, C and D). Likewise, *Irg1* shRNA significantly reduced ID8 ovarian carcinoma in the peritoneum (Figure 4, E and F). Although Irg1 targeting in pResMϕ had this profound effect, it did not alter the overall number of F4/80^+^ Mϕ recovered from the peritoneum (Figure 4G) or Gata6 expression by pResMϕ (Figure 4, H–J). Moreover, we confirmed that scrambled (Figure 4K) and *Irg1* (Figure 4L) shRNA vectors transduced pResMϕ similarly (see also Supplemental Figure 2A) and that EGFP encoded by our vectors was not detected in tumor cells or any host cells other than pResMϕ. Additionally, we observed no differences in cytokine gene expression in pResMϕ transduced with *Irg1* shRNA as compared with expression levels in the scrambled control (Supplemental Figure 2B). Although multiple metabolites were increased in tumor-bearing mice, knockdown of other metabolic enzymes, namely arginase 1 and lactate dehydrogenases A and B, did not affect tumor burden (Supplemental Figure 3). To complement the lentivirus-based approach, we also found that adoptive transfer of *Irg1^–/–^*, but not WT, pResMϕ into clodronate-depleted recipient mice resulted in a similarly significant reduction of peritoneal tumor burden (Figure 4M). The adoptively transferred donor cells comprised more than 90% of the recovered pResMϕ in recipient mice, and equivalent yields of total pResMϕ were obtained from mice receiving cells from WT and *Irg1^–/–^* mice (Supplemental Figure 4). Taken together, these data demonstrate that tumor-induced *Irg1* expression is required for pResMϕ-associated tumor promotion.

### Irg1 in pResMϕ regulates OXPHOS.

**Figure 5:**
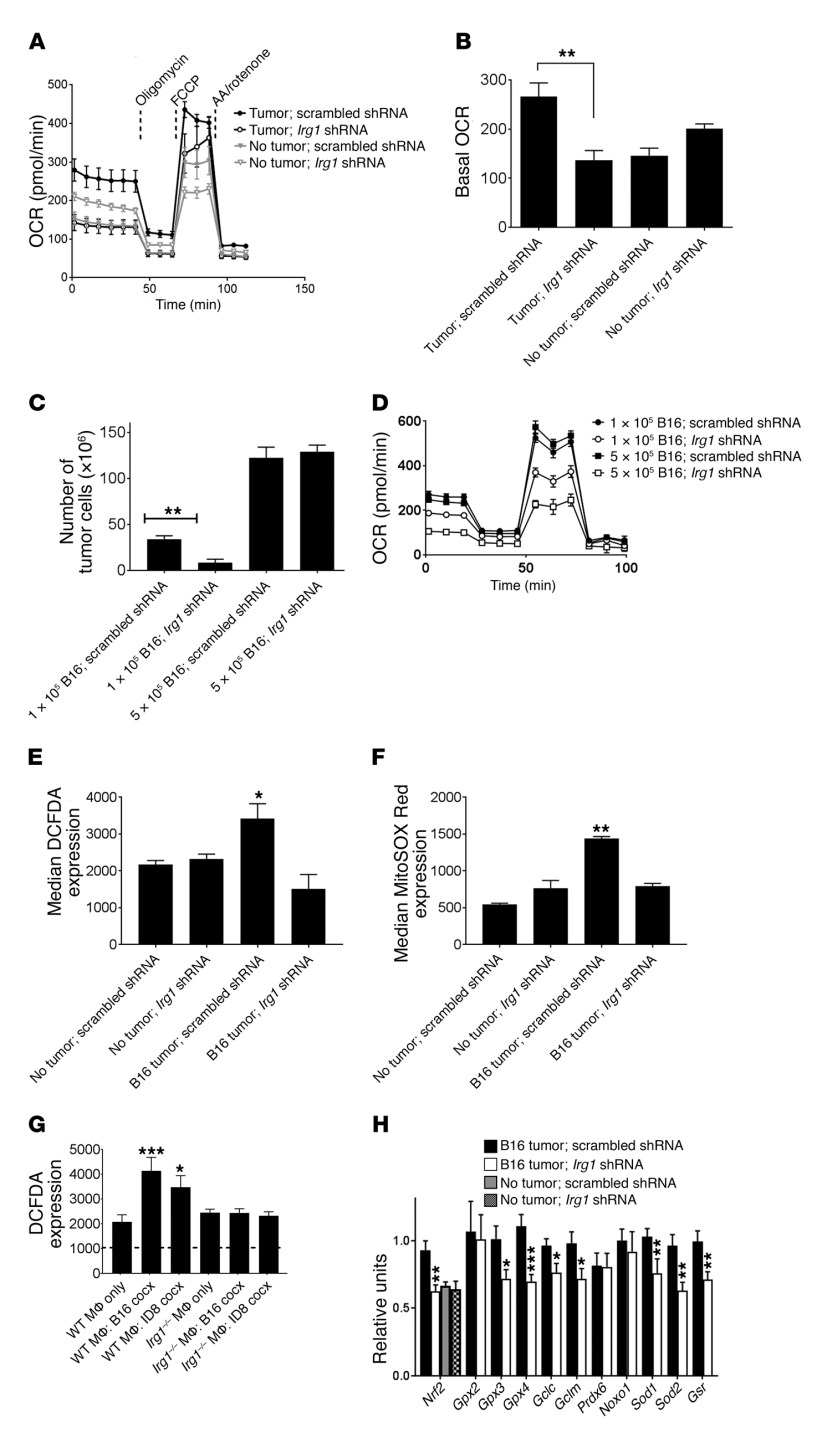
Itaconate regulates OXPHOS and mitochondrial ROS expression in pResMϕ. Extracellular flux was analyzed on F4/80-sorted pResMϕ from B16 tumor–bearing mice receiving either scrambled or *Irg1* shRNA (**A**). The OCR over time (**A**) and the basal OCR (**B**) were graphed. Data are representative of 3 experiments. **P < 0.01, by ANOVA with Tukey’s multiple comparisons test. Tumor burden (**C**) and pResMϕ extracellular flux (**D**) were evaluated in mice that received 1 × 10^5^ or 5 × 10^5^ B16 tumor cells and either scrambled or *Irg1* shRNA (*n* = 5). **P < 0.01, by ANOVA with Tukey’s multiple comparisons test. pResMϕ ROS production was assessed by measuring the median CM-H2DCFDA (**E**) and MitoSOX Red (**F**) expression by flow cytometry (*n* = 6). *P < 0.05 and **P < 0.01, by ANOVA with Tukey’s multiple comparisons test. (**G**) pResMϕ from WT or *Irg1^–/–^* mice were cocultured in vitro with the indicated tumor cells for 48 hours. Median CM-H2DCFDA expression was evaluated by flow cytometry (*n* = 3). The dotted line denotes no DCFDA control staining. *P < 0.05 and ***P < 0.001, by ANOVA with Tukey’s multiple comparisons test. (**H**) Gene expression levels modulated by oxidative stress and ROS in mice that received scrambled or *Irg1* shRNA were evaluated by qPCR (*n* = 14). *P < 0.05, **P < 0.01, and ***P < 0.001, by Mann-Whitney *U* test. Data represent the mean ± SEM.

Knowing that itaconic acid can regulate OXPHOS and cell metabolism in other systems (13, 18, 19), we next determined whether *Irg1* shRNA treatment alters pResMϕ metabolism and whether those changes could be important for the control of tumors. Treatment of tumor-bearing mice with *Irg1* shRNA reduced the basal and maximal OCRs of pResMϕ to levels comparable to those in non–tumor-bearing control mice (Figure 5, A and B). In contrast, we found that *Irg1* shRNA had no effect on the basal OCR of pResMϕ from control mice.

We reasoned that reduced OXPHOS by pResMϕ in *Irg1* shRNA–treated mice could either be due to direct regulation by resulting itaconic acid or an indirect result of the lower tumor burden in these mice. To distinguish between these 2 possibilities, we increased the tumor dose to a level at which *Irg1* shRNA treatment was no longer capable of reducing the tumor burden (Figure 5C). At this higher dose, however, we found that *Irg1* shRNA was still capable of reducing Mϕ basal and maximal OCRs (Figure 5D). Thus, the reduced OXPHOS following *Irg1* shRNA is not simply due to a reduced tumor burden but rather is a direct effect of the lack of *Irg1*.

### Itaconic acid regulates mitochondrial ROS production in pResMϕ.

Recent studies have shown ROS production to be a component of epithelial IRG1–mediated regulation of inflammation (24) and the antimicrobial properties of IRG1 in Mϕ lineage cells in a zebrafish model (23). Therefore, we sought to determine whether *Irg1* could modulate ROS levels in pResMϕ in the context of tumor regulation. ROS levels were evaluated by several different means. First, we used the cell-permeant dye CM-H2DCFDA that detects hydroxyl, peroxyl, and other ROS within cells. *Irg1* shRNA treatment significantly reduced the median expression of CM-H2DCFDA in pResMϕ to levels observed in non–tumor-bearing mice (Figure 5E and Supplemental Figure 5A). Recognizing that CM-H2DCFDA, as a general oxidative stress indicator, has certain limitations (36), we also measured MitoSOX Red as a marker of superoxide specifically attributable to mitochondrial function. We consistently found that *Irg1* shRNA treatment significantly reduced the median expression of MitoSOX Red to control levels (Figure 5F and Supplemental Figure 5B). Furthermore, we detected increased CM-H2DCFDA levels when pResMϕ from naive WT mice were cocultured in vitro with either B16 or ID8 tumor cells, but not when pResMϕ from *Irg1^–/–^* mice were used (Figure 5G and Supplemental Figure 5C). Changes in ROS production were further confirmed by analyzing the expression of 11 antioxidant genes whose expression is tied to ROS levels (37). By quantitative PCR (qPCR), we found that the expression of *Nrf2*, a key regulatory factor of antioxidant gene expression, as well as that of 7 of 10 other antioxidant genes was significantly downregulated in pResMϕ from *Irg1* shRNA–treated mice (Figure 5H). The levels of *Nrf2* expression in pResMϕ from *Irg1* shRNA–treated tumor-bearing mice were comparable to those of pResMϕ from non–tumor-bearing control mice. Extracellular ROS levels were significantly elevated in the peritoneal lavage fluid from tumor-bearing mice as compared with levels in fluid from control mice (Supplemental Figure 6A). Taken together, these data indicate that tumor-induced *Irg1* expression promotes mitochondrial ROS levels and subsequent antioxidant activity in pResMϕ. Since ROS have been reported to induce cytokine expression and pResMϕ can be important sources of ROS, the lack of changes in their cytokine gene expression in these pResMϕ in the context of changing ROS levels (Supplemental Figure 2B) was somewhat surprising. Our metabolomic data analysis revealed that pResMϕ have significantly higher amounts of glutathione as compared with bone marrow–derived Mϕ, which may partly protect them from such ROS-mediated effects (Supplemental Figure 6B).

### Itaconic acid–mediated ROS in pResMϕ regulate MAPK activation in tumor cells.

**Figure 6:**
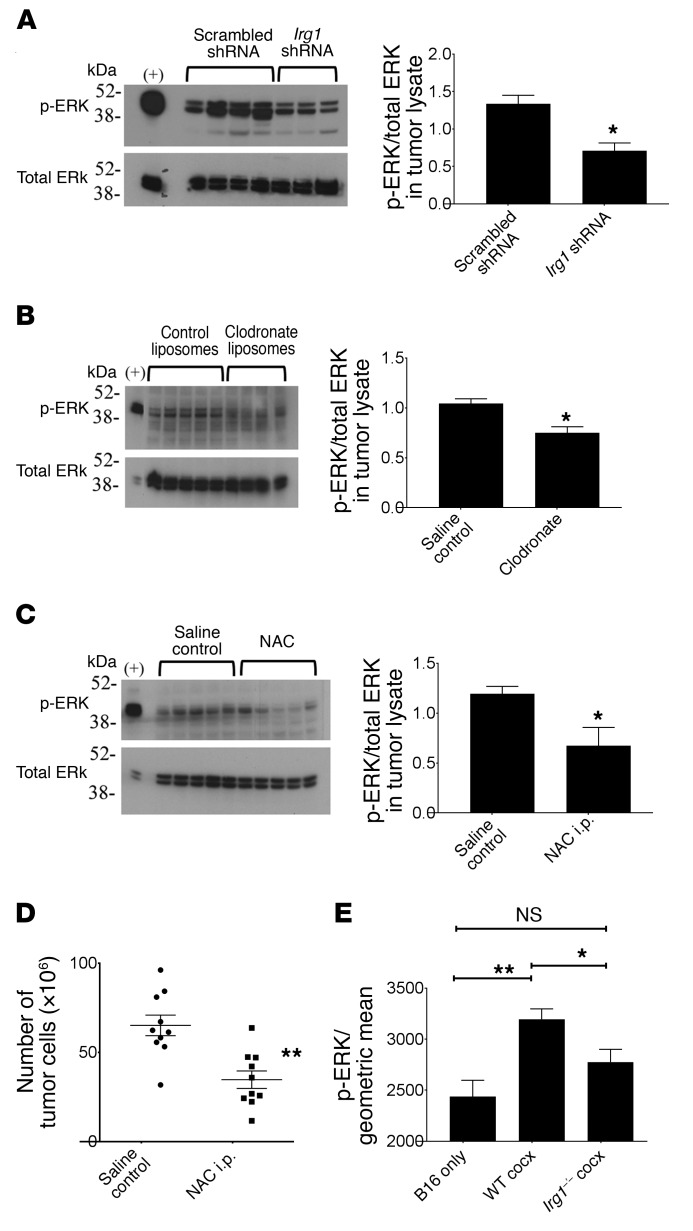
pResMϕ regulate MAPK activation in peritoneal tumors via itaconate and ROS. B16 tumor lysates were prepared as described in Methods and analyzed by Western blotting for expression of p-ERK and total ERK. (**A**) Tumor lysates from mice that received scrambled or *Irg1* shRNA constructs were compared (*n* ≥4). *P < 0.05, by Mann-Whitney *U* test. (**B**) Tumor lysates from mice that received control or clodronate liposomes were compared. *P < 0.05, by Mann-Whitney *U* test. (**C**) Tumor lysates from saline control– or NAC-treated mice were analyzed (*n* = 5). *P < 0.05, by Mann-Whitney *U* test. (**D**) NAC i.p. treatment reduced the number of B16 tumor cells (*n* = 10). **P < 0.01, by Mann-Whitney *U* test. (**E**) B16 tumor cells were cultured in vitro alone or in coculture with the indicated pResMϕ. After 48 hours, tumor cells, gated by CD146^+^F4/80^–^ expression, were analyzed by flow cytometry for intracellular p-ERK expression. *P < 0.05 and **P < 0.01, by ANOVA, corrected for multiple comparisons. Data represent the mean ± SEM.

We next sought to mechanistically link tumor-induced, *Irg1*-dependent Mϕ ROS levels with tumor promotion. Although ROS have well-described antitumor effects in some settings, it has also been shown to activate pathways promoting tumor growth (25, 28). We found that specific *Irg1* knockdown in pResMϕ significantly reduced the levels of phosphorylated ERK (p-ERK) in peritoneal B16 tumors (Figure 6A). We also found that clodronate-mediated removal of pResMϕ reduced p-ERK levels to a similar degree in peritoneal tumors (Figure 6B). Treatment of tumor-bearing mice with the antioxidant *N*-acetylcysteine (NAC) significantly reduced p-ERK levels in tumor lysates (Figure 6C) and the peritoneal tumor burden (Figure 6D). Furthermore, p-ERK was significantly elevated in B16 tumor cells cocultured in vitro with WT, but not *Irg1^–/–^,* pResMϕ (Figure 6E), and treatment of B16 and ID8 tumors with PD98059, a selective inhibitor of the MAPK cascade, significantly inhibited tumor cell proliferation (Supplemental Figures 6, C and D). Taken together, these findings suggest that tumor-induced *Irg1* and subsequent itaconic acid regulate peritoneal tumors, at least in part, through alterations in Mϕ-specific ROS.

### Expression of Irg1 in monocytes associated with human peritoneal tumors.

**Figure 7:**
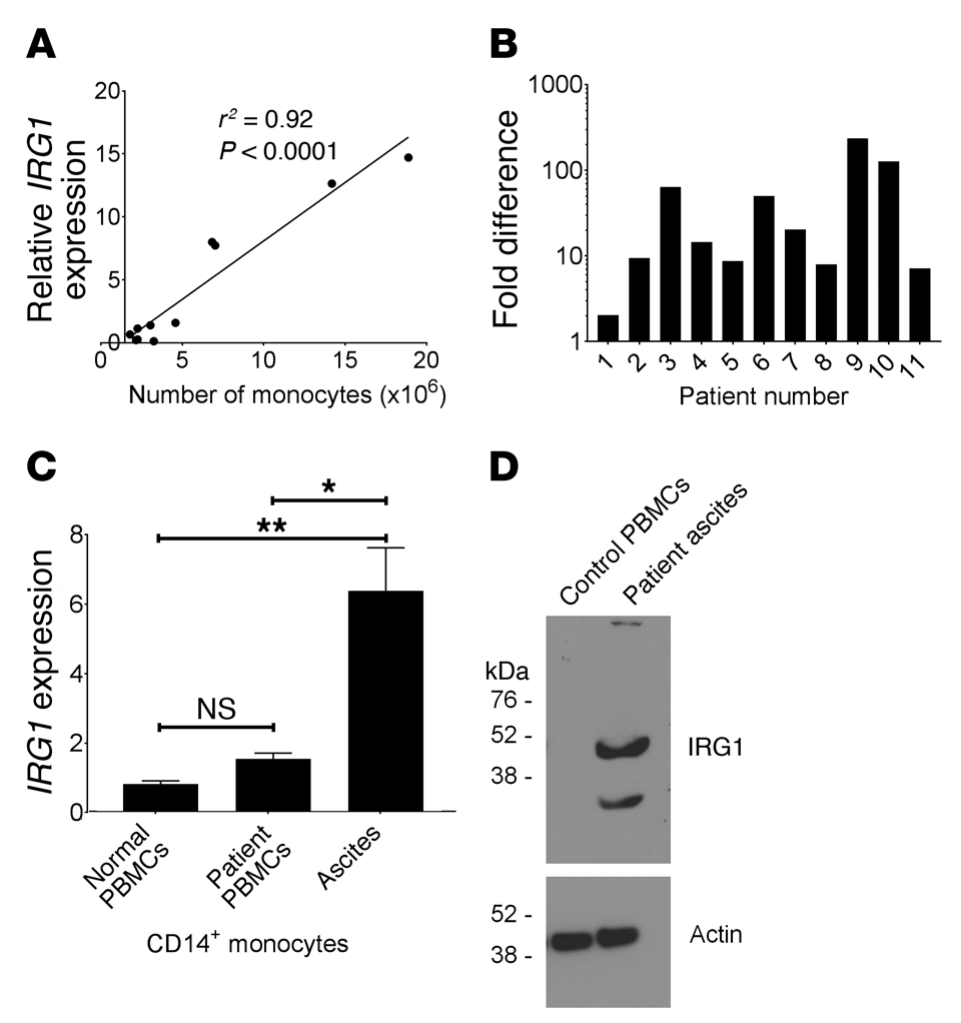
*IRG1* is expressed in monocytes associated with human peritoneal tumors. *IRG1* expression was evaluated by qPCR using total RNA isolated from cell fractions from 11 human patients with ovarian carcinoma. (**A**) *IRG1* expression levels were established by setting normal PBMC values to 1.0, and the relative levels in ascites monocytes were graphed in relation to the total number of CD45^+^CD14^+^ monocytes in each sample. Linear regression analysis was performed (GraphPad Prism) to obtain the best curve fit (*r^2^* = 0.93; *P* < 0.0001). (**B**) The fold difference in *IRG1* expression among monocytes and non-monocyte fractions is shown. CD14^+^ monocytes were purified as described in Methods. For each patient sample, the level of *IRG1* expression in the non-monocyte fraction was set to 1.0, and the relative level of *IRG1* expression in the corresponding monocyte fraction was graphed (log_10_). Significance was determined by a 1-sample Student’s *t* test using 1.0 as a theoretical mean (*P* < 0.05). (**C**) *IRG1* expression levels in CD14^+^ PBMCs isolated from 6 healthy volunteers and 5 patients were compared with *IRG1* levels in the 11 ascites samples. *P < 0.0 and **P < 0.01, by ANOVA corrected for multiple comparisons. (**D**) IRG1 protein expression in CD14^+^ monocytes isolated from healthy blood and patients’ ascites was analyzed by Western blotting.

Having established a paradigm whereby tumor-induced *Irg1* in pResMϕ results in fatty acid–mediated increases in ROS that in turn activate tumor cells in mice, we asked whether *IRG1* expression might be detected in human ovarian cancers. To that end, we isolated the cellular components from the peritoneal ascites fluid of 8 women with ovarian carcinoma and assayed for the expression of *IRG1*. In parallel, we assayed these samples for CD14^+^ monocytes by flow cytometry. We observed a significant association between the number of CD14^+^ monocytes in each bulk cell sample and *IRG1* levels, suggesting that these cells were expressing *IRG1* (Figure 7A). Consistent with that notion, we found that *IRG1* expression was significantly (*P* < 0.05) elevated in isolated monocytes from all 11 patients as compared with expression levels in non-monocyte cell fractions (Figure 7B). Furthermore, *IRG1* expression was significantly elevated in CD14^+^ monocytes isolated from patients’ ascites as compared with blood circulating monocytes from either healthy individuals or patients (Figure 7C). IRG1 protein was detected in CD14^+^ monocytes from patients with ovarian carcinoma, but not in normal peripheral blood mononuclear cells (PBMCs) (Figure 7D). These data suggest that myeloid cells associated with human ovarian cancers express IRG1, which positions them to promote tumor growth through itaconate production.

## Discussion

We show for the first time to our knowledge that itaconic acid is a critical metabolic component underlying the crosstalk between tumors and tumor-associated Mϕ. Itaconic acid levels were dramatically upregulated in pResMϕ from tumor-bearing mice, and this metabolite acts as a critical regulator of Mϕ metabolism, ROS production, and tumor progression, as shown in our study. *Irg1* expression was induced in pResMϕ by 6 cancer cell lines, including 3 different ovarian carcinoma cell lines. Our data expand on the importance of metabolic alterations in the tumor microenvironment and identify itaconate as a potential target for peritoneal cancer treatment. The upregulation of OXPHOS by tumor-promoting itaconate was highly localized, since inoculation of mice with tumors at distant sites did not increase the pResMϕ OCRs or ECARs, and coculture experiments revealed a cell-cell contact requirement for promotion of itaconate production. Itaconate production was the only metabolic pathway we tested that was capable of significantly regulating tumor burden. *Irg1* expression has been reported in several cell types (24, 38–40), but in the peritoneal cavity, it is *Irg1* expression by resident Mϕ that promotes tumor growth. It is possible that blood-borne cells enter the peritoneal cavity and take on the resident phenotype, however, our data indicate that infected pResMϕ remained over the course of the experiment. Although we could not directly examine the resident Mϕ population of patients with advanced cancer, *IRG1* expression in patients’ monocytes from tumor ascites suggests that this metabolite could be important in the progression of ovarian carcinoma.

Itaconic acid is emerging as an important regulator of Mϕ metabolism and effector function. The mechanisms by which tumors induce *Irg1* and itaconic acid remain unclear and warrant further investigation. *Irg1* induction was still observed when TNF-, IFN-γ-, IL-6-, or TLR4-KO mice were inoculated with tumors, however, we could not rule out the possibility that tumor-derived cytokines influence pResMϕ. One possibility may be the induction of hemoxygenase-1, a stress protein recently shown to induce *Irg1*, which may be associated with tumors and tumor-associated Mϕ (41, 42). Once upregulated, itaconic acid is accompanied by increases in fatty acid–driven OXPHOS. This appears to be in contradiction to the succinate dehydrogenase-inhibitory role ascribed to itaconic acid (18, 19). However, OXPHOS is principally driven by NADH recycling, which can be fueled through succinate dehydrogenase–independent mechanisms, such as the mitochondrial citrate and malate shuttles (43), which may operate differently in the presence of tumor. Our findings are consistent with those of Hall et al., who showed that *Irg1* depletion impaired fatty acid oxidation in Mϕ-lineage cells (23). Moreover, we found that upregulated itaconic acid in pResMϕ from tumor-bearing mice was associated with reduced levels of several long-chain fatty acids. Their oxidation may help fuel increases in OXPHOS that comprise a major source of cellular ROS (23, 44). Thus, itaconate is clearly important for the regulation of intracellular and extracellular ROS production in pResMϕ. ERK activation in tumor cells is probably one of many pathways activated by ROS that could potentially regulate tumor growth in vivo. We never detected *Irg1* expression in tumor cells, and if *Irg1* expression is relatively Mϕ specific, then targeting *Irg1* levels in the peritoneum may be a means to regulate pResMϕ-derived ROS without eliciting some of the side effects associated with systemic ROS inhibition.

Several lines of evidence support the hypothesis that pResMϕ, rather than newly migrated inflammatory monocytes, mediate the *Irg1*-mediated protumor effects. We administered lentiviral constructs 1 week prior to tumor inoculation, thus limiting metabolic programming before any tumor-mediated alterations in leukocyte populations could occur. Importantly, these infected Mϕ were labeled with EGFP, which remained with this Gata6^+^ cell population throughout our tumor studies. Thus, our alterations of itaconate production were specifically targeted to pResMϕ, with results consistent with our previous findings (3, 32). Therefore, pResMϕ specifically facilitate tumor progression in the peritoneum, in large part by tumor-mediated increases in itaconate production. It is becoming increasingly important to understand the regulation of itaconic acid expression in diverse Mϕ subsets. Since myeloid-derived suppressor cells (MDSCs), a heterogeneous population of tumor-associated Mϕ, utilize fatty acid oxidation to support their ROS production and immunosuppressive phenotype (45), it will be interesting to study metabolic changes in MDSC populations and determine whether targeting itaconic acid might regulate MDSC-associated ROS and MDSC-suppressive potential. Future studies aimed at elucidating the regulation of *Irg1* expression in myeloid cells of different anatomical compartments are warranted.

The identification of itaconic acid as an important regulator of pResMϕ function raises the intriguing question of how this might be used therapeutically for the control of ovarian and peritoneal tumors. Our results indicate that tumor burden is likely to be an important factor in dictating the success of IRG1-based therapies and that early intervention is important, since *Irg1* intervention was effective at lower, but not higher, tumor burdens. The importance of itaconic acid as an inhibitor of metabolic pathways such as those for isocitrate lyase in bacteria and glycolysis in mammals may yield important clues for its development as a therapeutic target. *Irg1*-deficient Mϕ have impaired bactericidal capabilities, and certain pathogenic bacteria, such as *Yersinia pestis* and *Pseudomonas aeruginosa*, degrade itaconate as a survival mechanism (46). Itaconate metabolism occurs via enzymes that convert itaconate into pyruvate and acetyl-CoA. Mammals have similar mechanisms for degrading itaconate (47) that are probably a means to counteract itaconate-mediated inhibition of glycolysis (48). Our data identify itaconic acid as a potential therapeutic target and support the need for additional research into the multitude of ways itaconate regulates tumor and host cellular responses at differing ratios between Mϕ and tumor burden levels so that the targeting of this metabolite for degradation becomes a potential means by which peritoneal tumors and perhaps other diseases can be treated.

## Methods

### Mice.

WT C57Bl/6 and CD45.1 (Ptprc^a^) mice were obtained from The Jackson Laboratory and bred at the NCI’s Frederick Cancer Research and Development Center. *Irg1^–/–^* mice were obtained from Michael Diamond (Washington University School of Medicine, St. Louis, Missouri, USA). Mice were used at 8 to 10 weeks of age, and female mice were used for ovarian carcinoma studies.

### Isolation of peritoneal Mϕ.

Peritoneal exudate cells were isolated by injecting 5 ml sterile PBS into the peritoneal cavity, gently massaging the anesthetized mouse, and slowly withdrawing the fluid. Mϕ contained within the exudate fluid were purified using biotinylated anti-F4/80 antibody (clone BM8; BioLegend) and magnetic bead separation (Miltenyi Biotec). Cells were counted using a Sysmex XP-300 (Roche Diagnostics).

### Cell lines.

B16 melanoma, 3LL, and MC38 cell lines were obtained from the American Type Culture Collection (ATCC). The murine ovarian surface epithelial cell lines MOSEC-ID8, IG10, and IF5 were obtained from Katherine Roby (University of Kansas Medical Center, Kansas City, Kansas, USA) and grown as described previously (49). All cell lines were confirmed to be free of mycoplasma contamination. Cells were propagated in DMEM supplemented with 10% FBS and L-glutamine. An in vivo–passaged ID8 cell line that could be reintroduced into naive mice was generated by injecting mice with 1 × 10^5^ ID8 cells and then collecting and centrifuging the resulting ascites fluid on approximately day 80 (50). For tumor cell–Mϕ coculture experiments, 5 × 10^5^ peritoneal Mϕ and tumor cells were mixed (1:1) and incubated in 6-well plates for 48 hours. In some experiments, tumor cells were seeded on Transwell inserts containing 3-μM pores (Costar). In some experiments, tumor cells were treated with the MAPK inhibitor PD98059 (50 μM; Cell Signaling Technology), and cell proliferation was quantified using the CellTiter Aqueous Non-Radioactive Cell Proliferation Assay (Promega).

### Isolation of human samples.

Peripheral blood leukocytes were isolated from healthy individuals and patients with ovarian carcinoma using Lymphocyte Separation Medium (Lonza). The ascites samples were centrifuged at 3,000 *g* for 10 minutes to remove the noncellular ascites fluid. Red blood cells were lysed by incubating the cell pellet in ammonium chloride potassium (ACK) lysing buffer for 10 minutes. Cells were washed with PBS and cryopreserved until use. Upon thawing, the cells were counted using a Sysmex XP-300 and analyzed by flow cytometry. The number of monocytes in each bulk cell fraction was determined by multiplying the total number of cells by the percentage of CD45^+^CD14^+^ cells (CD45 antibody clone HI30, CD14 antibody clone M5E2; BioLegend). In some experiments, monocytes were isolated using antibodies against CD14 and magnetically coupled beads (Miltenyi Biotec). Total RNA and cDNA used for qPCR analyses were prepared as described below.

### Lentiviral shRNA generation.

The In-fusion Cloning Kit (Clontech Laboratories) was used to modify the previously described pHR′SIN-cPPT-SEW plasmid (51). Lentiviral particles were produced by transfecting 293-T cells with lentiviral plasmid and helper constructs (52) (pCMV-Δ8.91 [GAG/POL, Tat and Rev] and pMD2.G [VSV-G coat]) using X-tremeGENE 9 Transfection Reagent (Sigma-Aldrich). Lentivirus was concentrated using the Lenti-X Concentrator Kit (Clontech) with supernatants from 293T cells 48 hours after transfection. Lentiviral particles were resuspended in PBS and stored at –80°C. Mice were injected i.p. 7 days prior to tumor cell injections (day –7) with 200 μl concentrated lentiviral particles.

### In vivo tumor model.

Mice were injected i.p. with 1 × 10^5^ tumor cells in 100 μl sterile saline. Some mice received 100 μl control or clodronate liposomes i.p. (clodronateliposomes.org) or 150 mg/kg NAC (Sigma-Aldrich) via the drinking water. Peritoneal lavages were done on day 9 unless otherwise indicated. For the adoptive transfer experiment, peritoneal lavage exudate cells from 10 WT or *Irg1^–/–^* mice were injected i.p. into clodronate-depleted, CD45 congenic mice 1 day prior to tumor inoculation. For quantitation of B16 melanoma cells, the tumors throughout the peritoneal cavity were surgically dissected, passed through Filtra-Bags (Labplas) and 100-μM nylon filters (Thermo Fisher Scientific), and the resulting single-cell suspension was counted on a Cellometer Auto T4 (Nexcelom Bioscience). For the in vivo–passaged ID8 tumor model, tumors were weighed and measured using calipers. Extracellular ROS contained within peritoneal lavage fluid was measured using the Amplex Red Hydrogen Peroxide/Peroxidase Assay Kit (Thermo Fisher Scientific).

### In vivo MRI.

The MRI experiments were conducted using a 3.0T Intera Achieva Clinical Scanner (Philips) equipped with a 40-mm-diameter solenoid receiver coil (Philips Research) to detect the initial point of the peritoneal tumor growth and monitor its progression over time.

A multislice, T2-weighted turbospin echo (T2w-TSE) imaging sequence was applied in the coronal direction with a field of view (FOV) of 50 × 30 × 12 mm to cover the mid-part of the abdominal area. To minimize motion-induced artifacts in the resulting image, a respiratory triggering technique was used. The images were obtained with a repetition time (TR) of 5333 ms, an echo time (TE) of 80 ms, an in-plane resolution of 0.180 × 0.180 mm, and a slice thickness of 0.5 mm. A fat suppression technique, spectral presaturation with inversion recovery (SPIR), was used to suppress the fat component in the images and thus to help distinguish fat from tumor tissue in the peritoneal cavity. The MRI methodology described generates high-resolution T2w images with a clear contrast between the tumor mass and the peritoneal organs and allows post-imaging analysis for a quantitative assessment of tumor burden.

### Primary metabolite analysis.

Magnetically sorted peritoneal Mϕ (5.0 × 10^6^ to 7.5 × 10^6^) were pelleted and snap-frozen in liquid nitrogen. Samples were further processed and analyzed for untargeted primary metabolites by the West Coast Metabolomic Center (UCD, Davis, California, USA). Mass spectrometric gas chromatography–time of flight (GC-TOF) analysis was performed using the Agilent GC 6890/LECO Pegasus III instrument. Samples were normalized using the sum of the peak heights for all identified metabolites. Metabolite data were analyzed using IPA.

### Extracellular flux analysis.

Magnetically sorted resident Mϕ were seeded at 1.0 × 10^6^ (24-well) or 0.3 × 10^6^ (96-well) cells per well in complete media (+0.5 μM retinoic acid, +20 ng/ml M-CSF) and incubated for 2 hours. Plated cells were washed gently with PBS and incubated with Seahorse Bioscience assay media supplemented with 2 mM glutamine and 25 mM glucose (+0.5 μM retinoic acid, +20 ng/ml M-CSF) for 1 hour at 37°C with no CO_2_. Extracellular flux analysis was performed at 37°C with no CO_2_ using the XF-24 or XF-96 analyzer (Seahorse Bioscience) according to the manufacturer’s instructions. Port additions and times were used as indicated in the figures.

### Flow cytometric analysis.

Cells (1 × 10^6^) were incubated for 15 minutes in cell-staining buffer (0.1% BSA, 0.1% sodium azide) containing 250 μg/ml 2.4G2 ascites. In some experiments, cells were incubated with 5 μM CM-H2DCFDA or MitoSOX Red mitochondrial Superoxide Indicator (Invitrogen, Thermo Fisher Scientific) for 20 minutes at 37°C. Cells were stained with fluorescently conjugated antibodies (F4/80 antibody clone BM8; CD49f antibody clone GoH3; Ly6C antibody clone AL-21; BD Pharmingen) for 20 minutes. For intracellular staining, surface-labeled cells were fixed and permeabilized (Invitrogen, Thermo Fisher Scientific) and incubated with Gata6 antibody (clone D61E4; Cell Signaling Technology) or p-ERK1/2 (Thr202/Tyr204) antibody (clone 4B11B69; BioLegend). After washing, the labeled cells were analyzed on an LSR II Flow Cytometer using FACSDIVA software (BD Biosciences).

### qPCR.

Total RNA was isolated using a High Pure RNA Isolation Kit (Roche Diagnostics). RNA was reverse transcribed using the High Capacity cDNA Archive Kit (Applied Biosystems). Genes of interest were examined by gene expression assays (Applied Biosystems). Briefly, 10 ng cDNA was placed into a final volume of 20 μl containing 10 μl TaqMan Universal PCR Mix (Applied Biosystems, Thermo Fisher Scientific) and 1 μl primer/probe gene expression assay. All samples were run on an ABI 7300 Real-Time PCR system and analyzed using the ΔΔCt method (53). Gene expression was normalized to levels of the housekeeping gene *HPRT*.

### Western blot analysis.

Purified monocytes and Mϕ were lysed in RIPA buffer. Tumors were homogenized in RIPA buffer using a GentleMACS Tissue Dissociator (Miltenyi Biotec). Total protein was quantitated by bicinchoninic acid assay (Thermo Fisher Scientific). Total protein (10–20 μg/lane) was separated by SDS-PAGE and transferred to PVDF membranes (Invitrogen, Thermo Fisher Scientific). Membranes were blocked for 1 hour with 5% BSA in TBS plus 0.05% Tween 20 and incubated overnight at 4°C with a primary antibody against IRG1 (Abcam), total or p-p44 or p-p42 MAPK (ERK1/2) (Cell Signaling Technology). Washed membranes were incubated with HRP-conjugated anti-mouse IgG secondary antibody and visualized using ECL (Invitrogen, Thermo Fisher Scientific).

### Statistics.

Statistical differences between groups were analyzed using GraphPad Prism software. A *P* value of less than 0.05 was considered statistically significant. The tests used include ANOVA with Tukey’s multiple comparisons test, Mann Whitney *U* test, Student’s *t* test.

### Study approval.

All mice were used in accordance with an approved NCI Frederick IACUC protocol. All patients provided written, informed consent at study enrollment. Peripheral blood was collected from 6 healthy individuals and 5 patients with ovarian carcinoma. Peritoneal ascites fluid was collected from 11 patients with ovarian carcinoma, who had fluid removed for therapeutic purposes, and deidentified. The NIH Office of Human Subjects Research determined that federal regulations for the protection of human subjects did not apply to this study, based on the Code of Federal Regulations, Title 45, Part 46.

## Author contributions

JMW designed and performed experiments and wrote the manuscript; LCD contributed to the interpretation of the results; MK performed experiments; LI performed experiments; MKO provided patients’ samples; RYSC provided reagents; LAR contributed to the interpretation of the results; CMA provided patients’ samples; DAW contributed to the interpretation of the results; DWM contributed to the interpretation of the results and wrote the manuscript.

## Supplementary Material

Supplemental data

## Figures and Tables

**Table 1:**
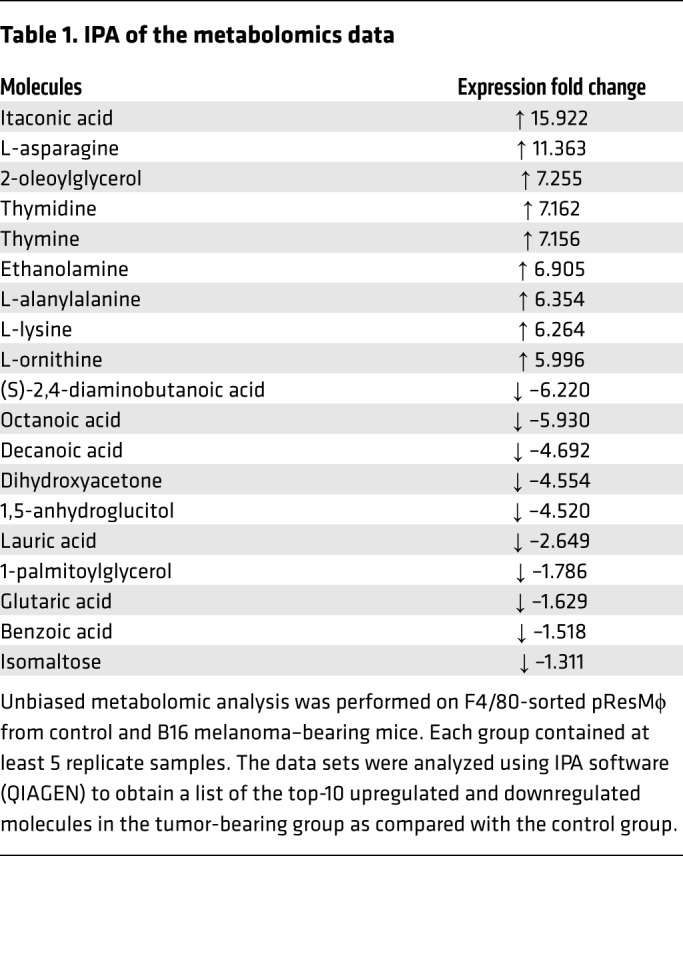
IPA of the metabolomics data

## Version 1 06/19/2018

In-Press Preview

## Version 2 07/30/2018

Electronic publication

## Version 3 08/31/2018

Print issue publication
