# Supplementary material for: Itaconic acid mediates crosstalk between macrophage metabolism and peritoneal tumors
Source: J Clin Invest. 2018 Jul 30;128(9):3794–805. doi: 10.1172/JCI99169 (PMC6118601; doi:10.1172/JCI99169)
Supplement: Supplemental data [file jci-128-99169-s052.pdf]

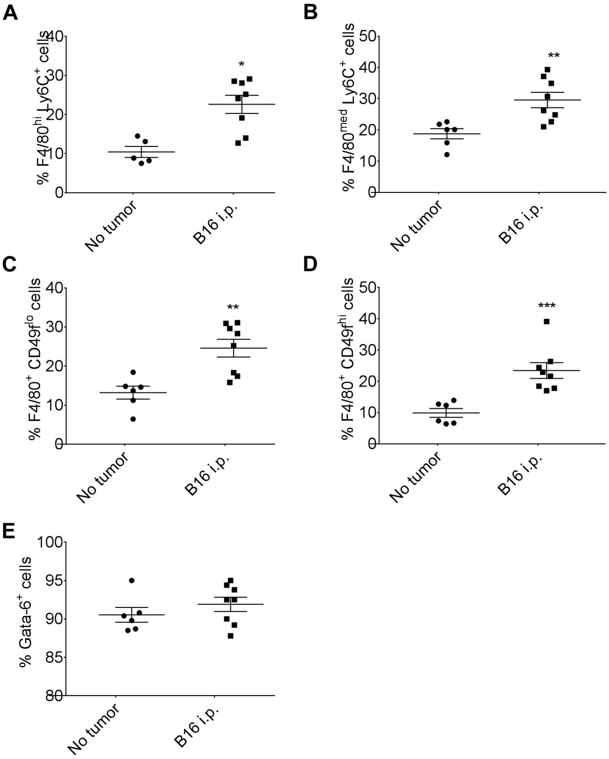

Supplemental Figure 1. Characterization of peritoneal leukocytes from tumor-bearing hosts. Flow cytometric analysis was performed on the peritoneal lavage cells from naïve and B16 tumor-bearing mice. The graphs depict the frequency of (A) F4/80^{hi} Ly6C^{+}, (B) F4/80^{medium} Ly6C^{+}, (C) F4/80^{+} CD49f^{lo} and (D) F4/80^{+} CD49f^{hi} cells in 6 samples (2 experiments each consisting of triplicate samples). (E) Among F4/80 sorted leukocytes, equivalent Gata-6 expression in pResMf from 6 non-tumor and 8 tumor-bearing mice was evaluated by intracellular flow cytometry.

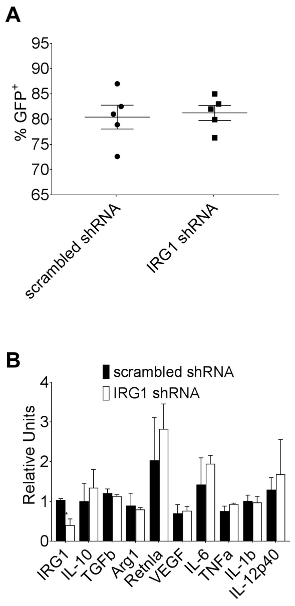

Supplemental Figure 2. Irg1 targeting does not alter the inflammatory gene signature of pResMϕ. (A) The percentage of eGFP$^+$ pResMϕ from mice receiving scrambled or *Irg1* shRNA is shown. (B) mRNA from F4/80 sorted pResMϕ from mice that received scrambled or *Irg1* shRNA was isolated and analyzed by qPCR. For each gene, one of the scrambled shRNA samples served as the 1.0 reference point (n=3; Student's unpaired t-test). The graph depicts mean ± SEM values.

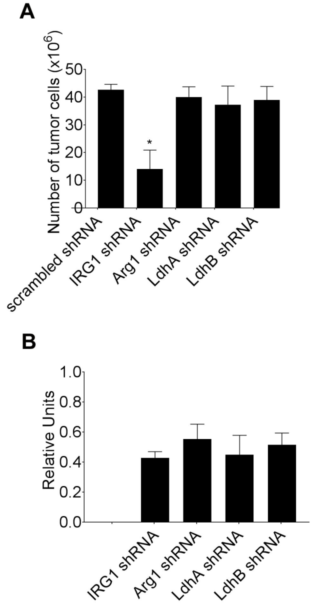

Supplemental Figure 3. Lentiviral targeting of other metabolic genes does not alter tumor burden. B16 tumor-bearing mice were treated with lentiviral shRNA (scrambled control or silencing the indicated genes). (A) B16 tumor burden was quantitated on day 9 (n=5; Mann Whitney U test). (B) The gene expression of *Irg1, Arg1, LdhA and LdhB* was evaluated by qPCR. For each gene, the expression levels in the scrambled shRNA control group was set to 1.0 and the relative levels are shown. The graphs depict mean ± SEM values.

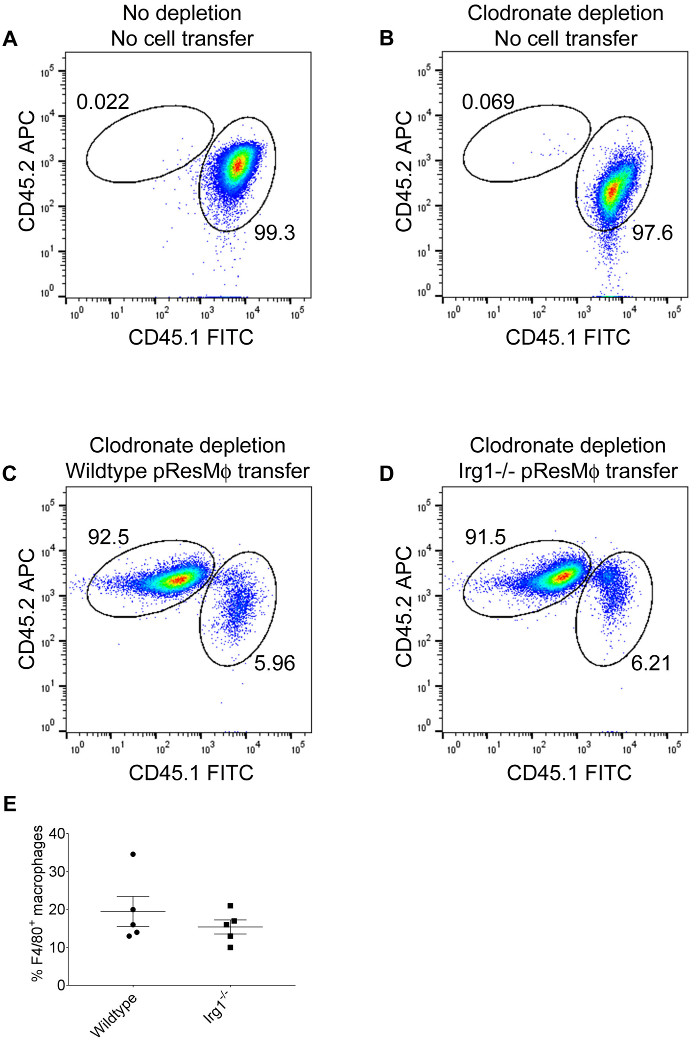

Supplemental Figure 4. Characterization of adoptively transferred pResMϕ. The clodronate-depleted, recipient mice were CD45.1 congenic mice and the donor mice of the wildtype or *Irg1*$^{-/-}$ peritoneal cells express CD45.2. (A) Flow cytometry confirmed that the recovered F4/80^{+} pResMϕ from control mice (in the absence of any cell transfer) were CD45.1^{+}. (B) Greater than 90% of the F4/80^{+} pResMϕ in adoptive transfer recipient mice were CD45.2^{+} donor cells. Results are representative of 5 mice per group. (C) Equivalent frequencies of F4/80^{+} pResMϕ were recovered from mice receiving wildtype or *Irg1*$^{-/-}$ donor cells (n=5).

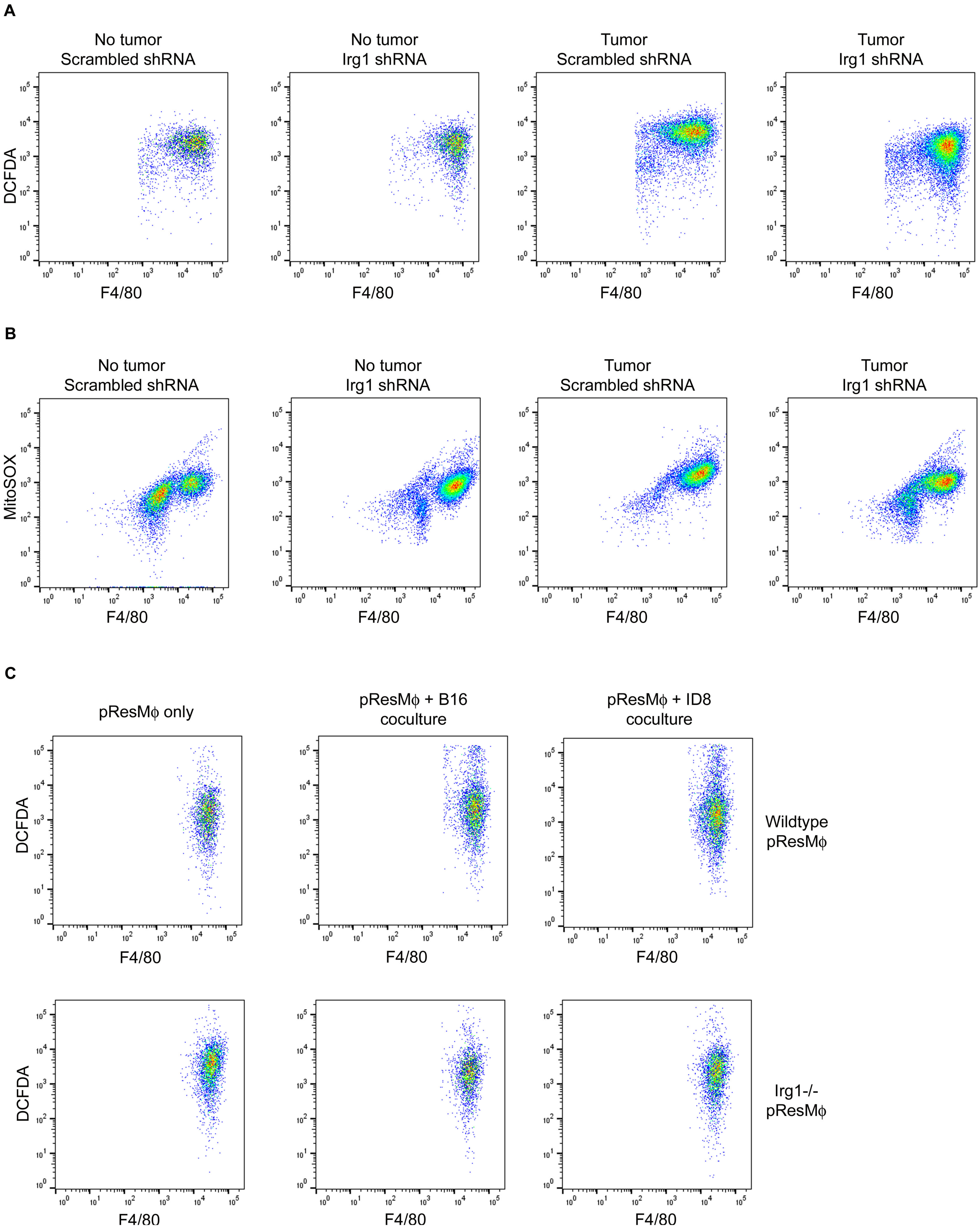

Supplemental Figure 5. IRG1 regulates mitochondrial ROS expression in pResMϕ. Flow cytometry scatter plots, representative of 6 mice, depict pResMϕ ROS production, as denoted by (A) DCFDA and (B) MitoSOX Red. (C) Flow cytometry scatter plots representative of 6 mice, depict DCFDA expression in pResMϕ from wildtype or *Irg1*$^{-/-}$ mice co-cultured *in vitro* with the indicated tumor cells for 48h. All FACS plots are gated on F4/80^{+} pResMϕ.

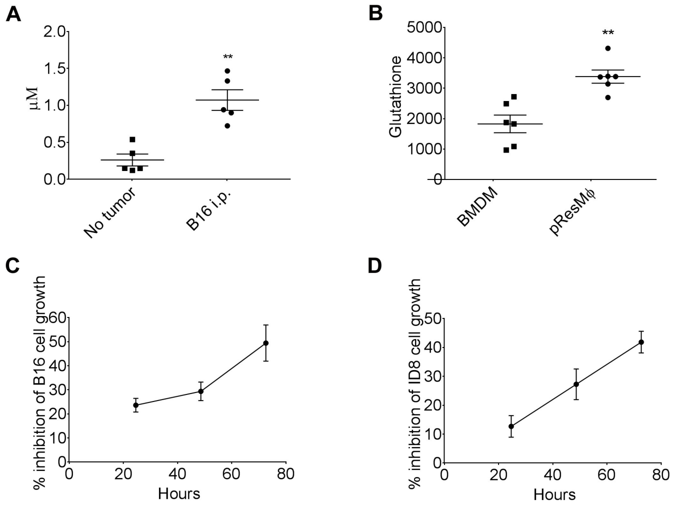

Supplemental Figure 6. Characterization of tumor-derived extracellular ROS and pResMϕ−associated glutathione expression. (A) Extracellular ROS in peritoneal lavages from naïve and tumor-bearing mice was analyzed using the Amplex Red Hydrogen Peroxide/Peroxidase Assay (n=5; Mann Whitney U test). (B) Glutathione levels between bone marrow-derived macrophages and pResMϕ were analyzed by metabolomics (n=6). (C) B16 and (D) ID8 tumor cells were grown *in vitro,* in quadruplicate wells, in the presence of the ERK inhibitor, PD98059 (50 μM) for the indicated times. The percent inhibition in cell proliferation was determined using an MTS assay according to the formula: ((OD_{490} control-OD_{490} treatment)/OD_{490} control)*100, with the OD_{490} for media alone subtracted from all values.
